# Hydrogen-rich water alleviates cadmium stress in Chinese flowering cabbage by enhancing antioxidant capacity, improving photosynthesis, and reducing cadmium uptake

**DOI:** 10.3389/fpls.2026.1890491

**Published:** 2026-08-10

**Authors:** Mengyao Liu, Cuinan Wu, Yufeng Chu, Hao Guo, Kai Cao, Tonghua Pan, Encai Bao, Keling Hu, Yi Zhang, Lin Ye, Wenjuan Xu

**Affiliations:** 1 School of Enology and Horticulture, Ningxia University, Yinchuan, China; 2 College of Horticulture, Shanxi Agricultural University, Jinzhong, China; 3 The Agriculture Ministry Key Laboratory of Agricultural Engineering in the Middle and Lower Reaches of the Yangtze River, Institute of Agricultural Facilities and Equipment, Jiangsu Academy of Agricultural Sciences, Nanjing, China; 4 College of Horticulture, Anhui Agricultural University, Hefei, China; 5 College of Agricultural Engineering, Jiangsu University, Zhenjiang, China

**Keywords:** antioxidant system, cadmium, Chinese flowering cabbage, HRW, uptake

## Abstract

Hydrogen-Rich Water (HRW) is a new environmentally friendly plant growth regulator that can effectively mitigate various abiotic stresses in plants. This study demonstrated that HRW significantly alleviated cadmium (Cd) stress in Chinese flowering cabbage. Compared to Cd treatment alone, Cd+HRW greatly enhanced the growth parameters and photosynthetic characteristics of the Chinese flowering cabbage. Under Cd+HRW stress, HRW notably reduced the malondialdehyde (MDA) content in shoots. Concurrently, HRW increased the activity of antioxidant enzymes, including peroxidase (POD), superoxide dismutase (SOD), ascorbate peroxidase (APX), and catalase (CAT), with Cd stress. And the levels of hydrogen peroxide (H_2_O_2_) and superoxide anion (O_2_^−^) generated by Cd stress in the Chinese flowering cabbage were significantly reduced by exogenous HRW. These findings collectively suggested that HRW boosts the antioxidant capacity of Chinese flowering cabbage under Cd stress. Additionally, the levels of nonenzymatic antioxidants such as ascorbic acid (AsA) and glutathione (GSH) were also remarkably improved by HRW. The detection results of Cd concentration revealed that Cd concentrations of shoot and root were significantly lowered by HRW in the Chinese flowering cabbage seedlings. However, the Cd transport coefficient was not significantly affected by HRW. This suggested that HRW-reduced Cd accumulation may result from inhibiting the uptake of Cd by HRW in Chinese flowering cabbage. To further validate this conclusion, we analyzed the expression levels of genes related to Cd uptake and transport in the Chinese flowering cabbage. The results revealed that HRW+Cd significantly downregulated the expressions of related genes (*BcIRT1*, *BcIRT2*, *BcNramp1*, and *BcZIP2*) involved in Cd uptake compared to Cd treatment alone in the Chinese flowering cabbage. However, the transcript level of the genes related to Cd transportation were not significantly affected by HRW. Therefore, based on the above experimental results, we suggested that HRW alleviated Cd stress may be on account of enhancing the antioxidant capacity and reducing the Cd uptake of the Chinese flowering cabbages.

## 1 Introduction

Chinese flowering cabbage (*Brassica campestris* L. ssp*. chinensis*) is a biennial herbaceous plant belonging to the Brassicaceae family. It is a variant of Chinese cabbage, uniquely developed through long-term domestication. In modern agriculture, the planting area and market share of Chinese flowering cabbage are growing. Based on the most recent agricultural statistics, the cultivation area for Chinese flowering cabbage in China has surpassed 1 million hectares, representing over 20% of the total leafy vegetable production (Zhou et al., 2016). However, as industrialization and urbanization advance, soil heavy metal pollution, especially for cadmium (Cd) contamination, has emerged as a significant environmental challenge (Zhang et al., 2017). A study has indicated that approximately 10% of farmland in the country suffers from varying degrees of Cd contamination, with up to 25% of vegetable fields being contaminated with Cd (Hu et al., 2020). Cd inhibits root development, thereby affecting the water and nutrient uptake of crops such as vegetables (Wang et al., 2020) Additionally, Cd interferes with the uptake and utilization of vital elements by plants, resulting in imbalanced nutrient levels. Moreover, Cd could unbalance photosynthesis, respiration, and protein synthesis in plants, causing symptoms such as leaf yellowing and shrinking, ultimately inhibiting plant growth and development (Rashid et al., 2023).

Cd is absorbed through the roots, not only affecting the quality and safety of vegetables but also posing a potential threat to human health (Alenyorege et al., 2018). Numerous studies have shown that leafy vegetables have the strongest accumulation capacity for Cd among all types of vegetables, being 2 to 3 times higher than root and tuber vegetables (Azeem et al., 2020; Rasool et al., 2020; Rasool et al., 2021). Hence, investigating effective strategies to control and reduce Cd levels in vegetables is crucial. At present, a variety of exogenous substances have the capacity to reduce Cd concentration in the edible portions of vegetables. For instance, exogenous melatonin notably decreases Cd concentration in the edible parts of alfalfa (*Medicago sativa* L.) and inhibits Cd-induced chlorophyll degradation as well as improves the antioxidant capabilities (Gu et al., 2017). Root application of humic acid (HA) could form HA-Cd complexes that are retained in the roots of lettuce. It reduced the movement of Cd to the shoot, which finally decreased the Cd content in the edible portions of lettuce (Haghighi et al., 2013). Under Cd stress, plants activate an antioxidant defense system composed of enzymatic and non-enzymatic components to scavenge excess ROS and maintain cellular redox homeostasis, thereby alleviating oxidative damage (Luo et al., 2024). However, it is noteworthy that although Cd stress upregulates the transcription of antioxidant enzyme genes, it often inhibits their enzyme activities. Therefore, plants may need to rely on specific signaling pathways, such as the NH_4_^+^-ABA-SAPK9-bZIP20 module, to ensure the activities of these antioxidant enzymes, ultimately enhancing plant tolerance to Cd stress (Di et al., 2024). Exogenous 5-aminolevulinic acid (ALA) helps maintain redox balance by safeguarding the photosynthetic system and inhibiting the expression of genes related to Cd transport, thereby reducing Cd content in the shoots of Chinese cabbage seedlings and alleviating Cd stress (Yang et al., 2022). The application of AsA reduces cadmium accumulation in spinach shoots while increasing the activity of the antioxidant enzyme system in spinach, which mitigates the growth inhibition induced by Cd stress (Javed et al., 2024). Indolebutyric acid (IBA) enhances photosynthesis and improves ROS scavenging ability, promotes Cd fixation in root cell walls, and reduces Cd translocation to the edible portions, which in turn improves the growth of lettuce under Cd stress (Chen et al., 2024). Choline inhibits Cd uptake and long-distance translocation, thus reducing the Cd accumulation in leaves and roots of tomato seedlings and maintaining the antioxidant system by reducing ROS production (Akpinar and Cansev, 2024).

In recent years, hydrogen (H_2_) has not only garnered attention for its benefits to human health but has also exhibited a role in regulating plant growth and metabolism when subjected to abiotic stress. Currently, plants primarily obtain H_2_ from HRW^[^ (An et al., 2021; Hu et al., 2021). For example, studies have shown that the contents of melatonin, gibberellin (GA), and indole-3-acetic acid (IAA) are induced by HRW treatment, which delays the senescence of okra (*Hibiscus esculentus* L.) fruit (Dong et al., 2023). Additionally, HRW effectively reduces nitrate, ROS, and MDA accumulation, which in turn improves the growth of Chinese cabbage seedlings (Wei et al., 2021). H_2_ also plays a crucial role in regulating root development and stress responses in plants. Specifically, HRW alleviates salt stress in cucumber seedlings by promoting the expression of genes involved in encoding antioxidant enzymes such as SOD, CAT, and POD, thus reducing oxidative damage under salt stress (Yu et al., 2023). In particular, when dealing with heavy metals like Pb and Cd, it has been demonstrated that HRW could modulate antioxidant defense responses, thus alleviating the inhibitory effects of their toxicity on rcould also (Cui et al., 2013; Ma et al., 2021). HRW also could reduce oxidative damage in alfalfa (*Medicago sativa* L.) seedlings induced by Cd toxicity, with enhancing sulfur compound metabolism and maintaining nutrient homeostasis (Dai et al., 2017). The expressions of genes involved in GSH metabolism and stimulates antioxidant activation are increased by the presence of HRW, which in turn enhances GSH metabolism and increases Cd tolerance in alfalfa (Cui et al., 2020). *IRT1*, *IRT2*, *ZIP2*, and *Nramp1*, which function as plasma membrane transporters facilitating the initial influx of Cd^2+^ from the rhizosphere into root cells (Wu et al., 2020; Wu et al., 2021; Tao and Lu, 2022). For translocation from roots to shoots, *HMA2* and *HMA4* encode P1B-type ATPases responsible for loading Cd into the xylem, whereas *HMA3* mediates vacuolar sequestration to detoxify cytosolic Cd (Antony et al., 2020). Furthermore, *ABCC1* and *ABCC2* serve as pivotal *ABC* transporters involved in the tonoplast transport of Cd-phytochelatin complexes, constituting a crucial detoxification mechanism (Park et al., 2012). Considering their distinct and indispensable roles in Cd uptake, translocation, and compartmentation, profiling the expression of these genes offers direct mechanistic insights into how HRW orchestrates Cd distribution and accumulation in plants.

Above all the research background, we assessed the influence of exogenous HRW on the growth, photosynthesis, antioxidant enzyme activities, MDA and ROS levels, nonenzymatic antioxidant systems, Cd content, and expressions of key genes in Chinese flowering cabbage under Cd stress. The findings indicated that HRW can improve the growth of Chinese flowering cabbage under Cd stress by enhancing photosynthesis, antioxidant enzyme activity, and the content of nonenzymatic antioxidant substances, as well as by restraining the expression of genes involved in Cd uptake, which ultimately results in lower Cd levels in the edible portions and alleviation of Cd stress in Chinese flowering cabbage.

## 2 Materials and methods

### 2.1 Plant materials, growth conditions, and treatments

The study used a common Chinese flowering cabbage (*Brassica campestris* L. ssp. *chinensis*) cultivar, ‘Qing Ye Jian Xin’. Seeds were sterilized with 75% ethanol for 60 s, followed by 15% NaClO for 5–10 min, and then rinsed five times with distilled water (30 s per rinse) to remove residual NaClO. After sterilization, the seeds were soaked in sterile water at room temperature for 5 h. The seeds were then placed on moist growth dishes and incubated at 24–27 °C for 24 h to induce germination. Once radicles emerged, uniform seedlings were selected and transferred to a culture container with distilled water. After 24 h of light exposure, the seedlings were moved to a vessel containing 1/4 Hoagland’s nutrient solution (pH 6.0). Growth conditions were as follows: photoperiod: 16/8 h (light/dark), temperature: 26/23 °C (day/night), light intensity: 200 μmol·m^−2^·s^−1^. Nutrient solution was refreshed on a daily basis.

When the seedlings had two true leaves, four treatment groups were established: the control group (CK, 1/4 Hoagland nutrient solution), the hydrogen-rich water treatment group (HRW, 50% hydrogen-rich water + nutrient solution), the cadmium treatment group (Cd, 50 μM CdCl_2_ (Sigma-Aldrich) + nutrient solution), and the combined treatment group (Cd+HRW). The seedlings were treated for 6 days, during which the treatment solutions were replaced daily with fresh solutions to maintain stable concentrations. After 6 days of treatment, physiological indicators and metal element contents were measured.

### 2.2 Measurement of growth parameters

Plant height was measured from the root-shoot junction to the apex using a ruler (0.1 cm precision). Root length was determined after gently washing off the nutrient solution and blotting with filter paper. Shoots and roots were separated at the cotyledon node, weighed immediately on an analytical balance (0.0001 g) for fresh weight (Wu et al., 2020; Yang et al., 2022).

### 2.3 Photosynthesis parameters and chlorophyll content measurement

Fresh leaf samples (0.2 g) were grounded and extracted in 25 mL of 95% ethanol in the dark for 12 h, following a previously described method (Li et al., 2022). The absorbance of the extract was measured at 665, 649, and 470 nm, and chlorophyll content was calculated using the following equations:


$$Chlorophyll\;a\;\left(Chl\;a\right)=13.95\;\times \;OD_{665}-\;6.88\;\times \;OD_{649}$$

$$Chlorophyll\;b\;\left(Chl\;b\right)=24.96\;\times \;OD_{649}-\;7.32\;\times \;OD_{665}$$

$$Carotenoids\;\left(Car\right)\;=\;\left(1000\;\times \;OD_{470}-\;2.05\;\times \;Chl\;a\;-\;14.8\;\times \;Chl\;b\right)/245$$

The photosynthetic parameters, such as stomatal conductance (*G*_s_), transpiration rate (*T*_r_), intercellular CO_2_ concentration (*C*_i_), and net photosynthetic rate (*P*_n_), were assessed using a photosynthesis system (Li-6800, LI-COR Inc., Lincoln, USA) from 8:30 am to 11:30 am.

### 2.4 MDA content measurement

MDA content determination was carried out following a previously described method (Gill and Tuteja, 2011). Fresh root samples, each 0.2 g, were homogenized in 5 mL of a solution comprising 10% trichloroacetic acid (TCA) and 0.25% thiobarbituric acid (TBA). The homogenate was placed in a 97 °C water bath for 30 min, followed by cooling in an ice bath. This was then centrifuged at 10,000 rpm for 20 min to obtain a supernatant. The absorbance of this supernatant was measured at wavelengths of 450 nm, 532 nm, and 600 nm. The MDA concentration was calculated using the equation: C = 6.45 × (OD_532_ - OD_600_) - 0.56 × OD_450_.

### 2.5 Measurement of active oxygen species H_2_O_2_ and O_2_^−^ contents

The content of O_2_^−^ is determined using spectrophotometry. Fresh root samples, each weighing 0.5 g, were homogenized in 3 mL of 65 mM phosphate buffer (pH 7.8). The homogenate was then centrifuged at 5800 rpm for 10 min. One ml of the resulting supernatant was mixed with 0.1 mL of 10 mM hydroxylamine hydrochloride and 0.9 mL of 65 mM phosphate buffer (pH 7.8). This mixture was incubated at 25 °C for 20 min, and the absorbance was measured at 530 nm (Ren et al., 2021).

The content of H_2_O_2_ is determined using spectrophotometry. Fresh root samples, each weighing 0.2 g, were homogenized in 5 mL of 50 mM phosphate buffer (pH 7.8). The homogenate was centrifuged at 10,000 rpm for 15 min at 4 °C. Titanium chloride (TiCl_4_) was added to the supernatant, and the mixture was incubated at room temperature for 10 min. The absorbance of the solution was then determined at 410 nm (Wahab et al., 2023).

### 2.6 Histochemical staining analysis

Root was stained with Schiff reagent, nitro blue tetrazolium, or DAB (3,3’-diaminobenzidine) for lipid peroxidation, O_2_^−^ or H_2_O_2_ measurement, respectively (Wahab et al., 2023). After staining, the plants were washed and observed using a Nikon Z 7II camera.

### 2.7 Antioxidant enzyme activity measurement

Fresh root samples, each 0.5 g, were homogenized on ice in 5 mL of 50 mM phosphate buffer (pH 7.8). The resulting homogenate was centrifuged at 12,000 rpm for 20 min at 4 °C. The supernatant was subsequently used for measuring enzyme activity and protein content.

The activities of SOD and POD enzymes were measured using spectrophotometry. One unit (U) of SOD activity was defined as the amount of enzyme that causes a 50% inhibition of NBT reduction, with the absorbance measured at 560 nm. The POD activity was determined using the guaiacol method, where the absorbance was measured at 470 nm, and the molar extinction coefficient used was 26.6 mM^−1^ cm^−1^. CAT activity was assessed by measuring the rate of H_2_O_2_ consumption over a 3-minute period at 240 nm, with a molar extinction coefficient of 39.4 mM^−1^ cm^−1^ (Hasanuzzaman et al., 2011). APX activity was quantified by monitoring the changes in absorbance due to AsA oxidation at 290 nm (Zhao et al., 2022).

### 2.8 Measurement of nonenzymatic antioxidant substances

AsA content was determined following a previously described method (Ren et al., 2021). Fresh root samples, each weighing 0.2 g, were first homogenized with 4 mL of TCA (trichloroacetic acid). The homogenate was then centrifuged at a speed of 12,000 rpm for 25 min at 4 °C.

A 0.2 mL of the supernatant was taken and mixed with a series of reagents for the determination of AsA content. These reagents included 0.75 mL of 5 mM EDTA (ethylenediaminetetraacetic acid) dissolved in 150 mM phosphate buffer at pH 7.8, 0.5 mL of 10% (v/v) TCA, 0.5 mL of 44% (v/v) phosphoric acid, and 0.15 mL of 0.3% (w/v) ferric chloride (FeCl_3_). This mixture was then centrifuged at 12,000 rpm for 20 min at 4 °C.

After centrifugation, 0.1 mL of the supernatant was taken and mixed with 0.9 mL of 150 mM phosphate buffer (pH 7.8) and 2 mL of 1 mM DTNB (5,5’-dithiobis (2-nitrobenzoic acid)). This final mixture was allowed to incubate for 10 min, after which the absorbance was measured at a wavelength of 412 nm to quantify the AsA content.

### 2.9 Measurement of metal element contents

Chinese flowering cabbage samples that had grown uniformly were submerged in a 20 mM EDTA-Na_2_ solution for 30 min. Following this treatment, the samples were rinsed thoroughly with deionized water to remove any residual solution. The samples were then segregated into their shoots and roots, carefully placed into individual envelopes, and subjected to a two-step drying process. Initially, they were dried at 150 °C for 30 min, followed by a reduction in temperature to 85 °C, where they were left until they reached a constant weight (Xiong et al., 2022). The weight of each sample was recorded; 15 plants per treatment with three replications.

Once dried, the samples were prepared for metal ion analysis by digesting them in 100% concentrated nitric acid using a microwave oven for one hour. After digestion, the samples were diluted with deionized water to a final volume of 50 mL, then filtered to remove any undigested particulate matter. A 10 mL aliquot of the filtered solution was taken for determining the content of metal ions, employing inductively coupled plasma optical emission spectrometry (ICP-OES, model Optima 2100DV, manufactured by PerkinElmer, located in San Jose, CA, USA).

### 2.10 Real-time quantitative RT-PCR analysis

Root RNA was extracted using the Vazyme FastPure Universal Plant Total RNA Isolation Kit, and RNA purity was evaluated by the 260/280 ratio (1.9–2.0). RNA was reverse transcribed to cDNA using the FastPure Universal Plant Total RNA Isolation Kit (Vazyme). DNA digestion and RNA reverse transcription were performed using the Vazyme HiScript III RT SuperMix for qPCR (+gDNA wiper) kit. The reverse transcription products were diluted to 100 μL, stored at -20 °C, and used for subsequent qRT-PCR analysis.

Quantitative real-time PCR (qRT-PCR) was conducted using a quantitative PCR instrument. The reaction mixture contained 2 μL of cDNA template and 18 μL of Bestar SybrGreen reagent. The expression levels of the target genes were normalized to the Actin gene using the 2^−ΔΔCt^ method (Tu et al., 2024). The sequences of the primers used for the qRT-PCR are provided in the appendix.

### 2.11 Statistical analysis

Experimental data are expressed as the mean ± standard deviation (SD). Each experiment was independently repeated at least three times with consistent results. For growth and physiological measurements, each treatment consisted of 15 seedlings per replicate with three biological replicates (n = 10), and data from individual plants were presented as scatter points overlaid on the bar graphs. For Cd content analysis, 15 plants per treatment were used with three replications. For qPCR analysis (Section 2.9), three independent biological replicates were performed, each with three technical replicates (n = 3). All data were analyzed for variance and significance using SPSS 20 statistical software. Differences among treatments were assessed by one-way analysis of variance (ANOVA) followed by the least significant difference (LSD) test at p < 0.05.

## 3 Results

### 3.1 The effect of HRW treatment on the growth of Chinese flowering cabbage under Cd stress

**Figure 1:**
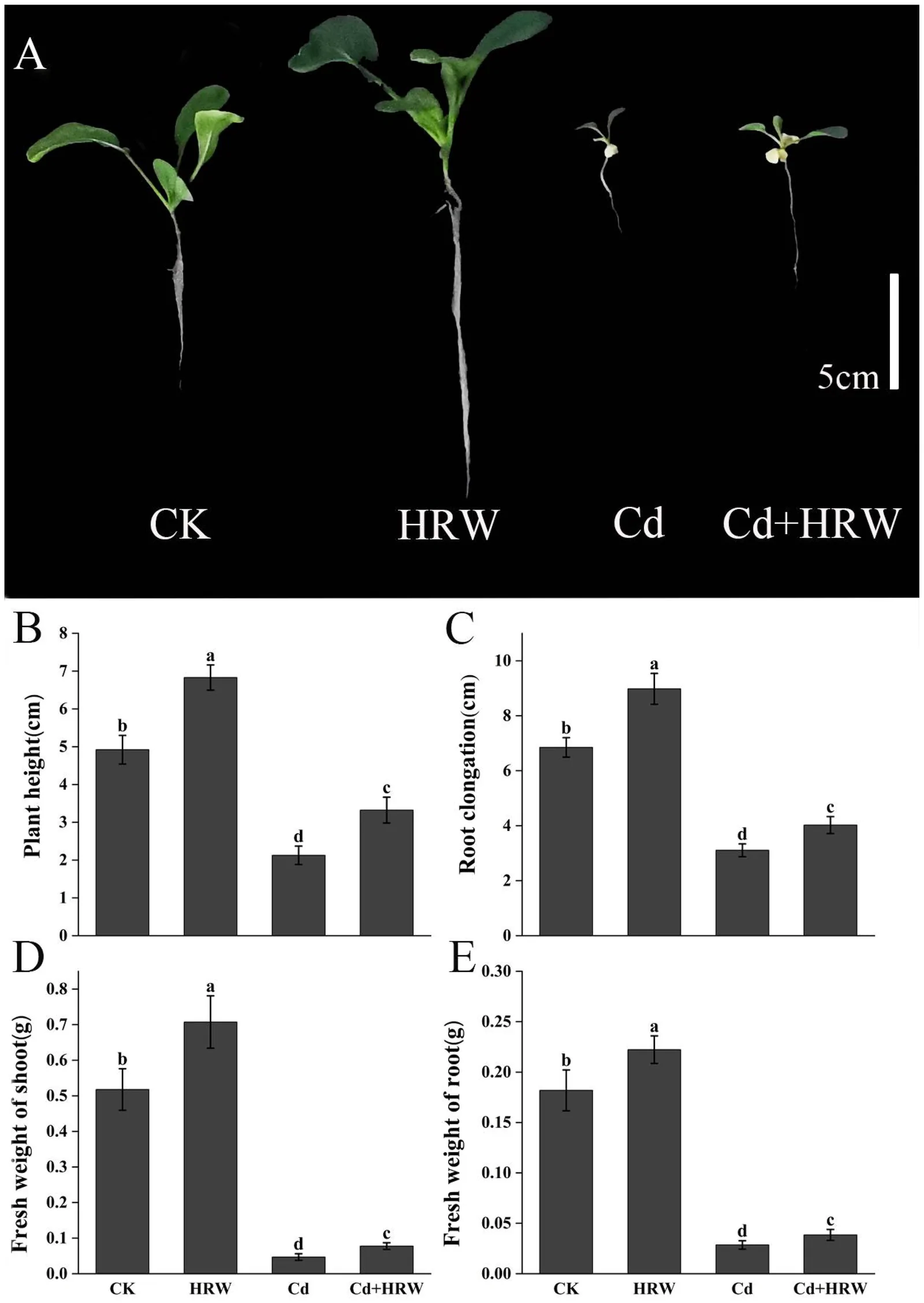
Effects of HRW treatment on the growth of Chinese flowering cabbage under Cd stress: **(A)** Growth phenotype of Qingye with different treatments, **(B)** changes in plant height, **(C)** changes in root length, **(D)** changes in shoot fresh weight, **(E)** changes in root fresh weight. Values are expressed as the mean ± standard error (n = 10). Columns marked with different letters indicate significant differences based on the LSD test (p < 0.05).

As shown in **Figure 1A**, compared with the CK, the sole application of HRW significantly promoted the growth of Chinese flowering cabbage seedlings, while Cd treatment markedly inhibited it, with reductions in plant height (**Figure 1B**), root length (**Figure 1C**), shoot fresh weight (**Figure 1D**), and root fresh weight (**Figure 1E**) compared to the CK group. The exogenous addition of HRW under Cd stress significantly alleviated this growth inhibition. Compared to the Cd treatment alone, the Cd+HRW treatment increased plant height, root length, shoot fresh weight, and root fresh weight by 24.33%, 13.42%, 6.07%, and 7.64%, respectively. Compared with the Cd treatment alone, the Cd+HRW treatment significantly alleviated this Cd-induced growth inhibition.

### 3.2 The effect of HRW on the photosynthetic performance of Chinese flowering cabbage under Cd stress

**Figure 2:**
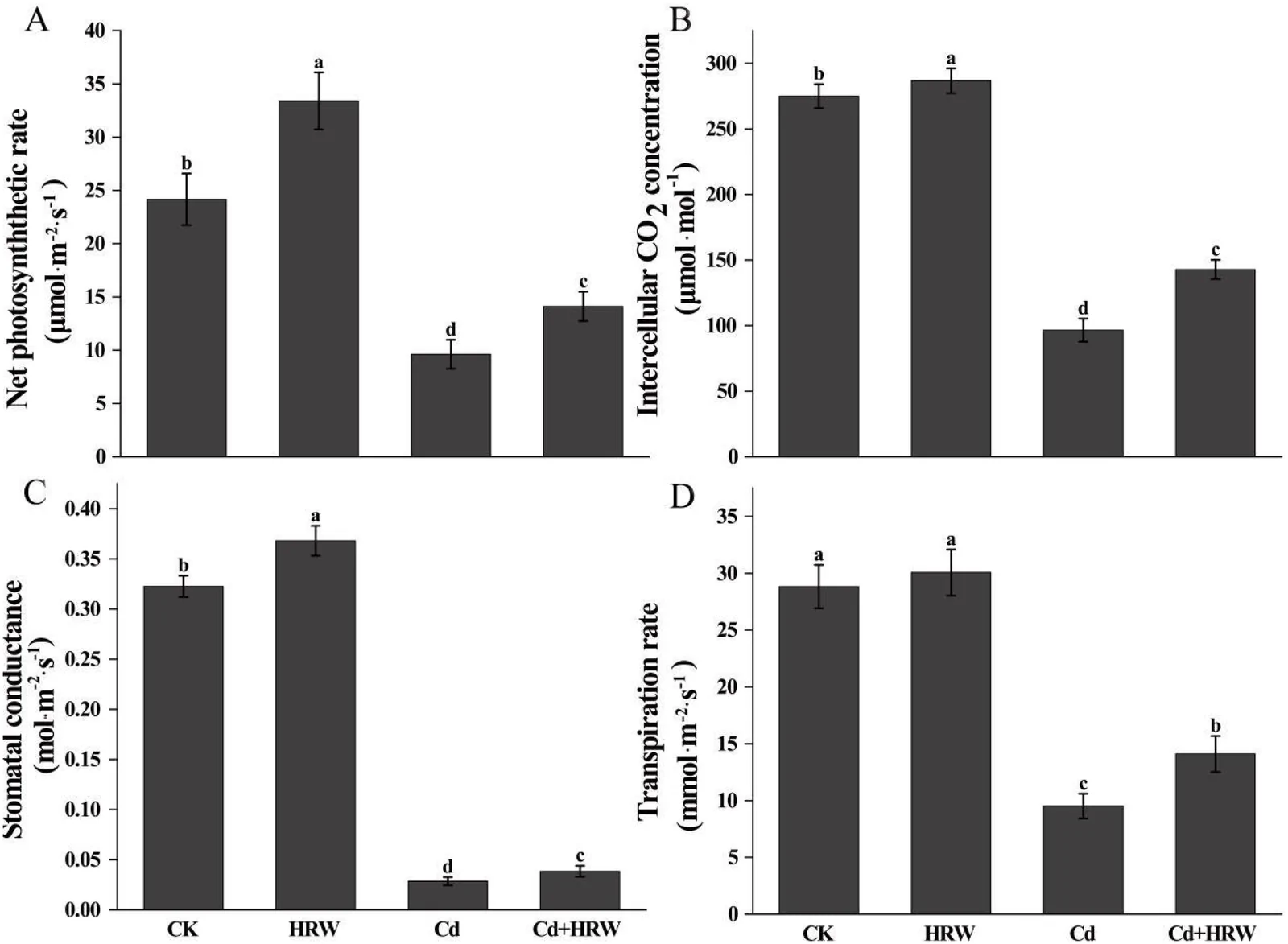
Effects of HRW treatment on photosynthesis of Chinese flowering cabbage under Cd stress: **(A)** changes in net photosynthesis, **(B)** changes in intercellular CO_2_ concentration, **(C)** changes in stomatal conductance, **(D)** changes in transpiration rate. Values are expressed as the mean ± standard error (n = 10). Columns marked with different letters indicate significant differences among treatments based on the LSD test (p < 0.05).

Compared with the Cd treatment, the exogenous addition of HRW significantly alleviated Cd-induced inhibition of photosynthesis. The net photosynthetic rate (**Figure 2A**), intercellular CO_2_ concentration (**Figure 2B**), stomatal conductance (**Figure 2C**), and transpiration rate (**Figure 2D**) of the Cd+HRW treatment increased by 19.11%, 16.21%, 3.21%, and 11.12%, respectively, compared with the Cd treatment. It indicated that HRW effectively improved photosynthetic performance under Cd stress.

**Figure 3:**
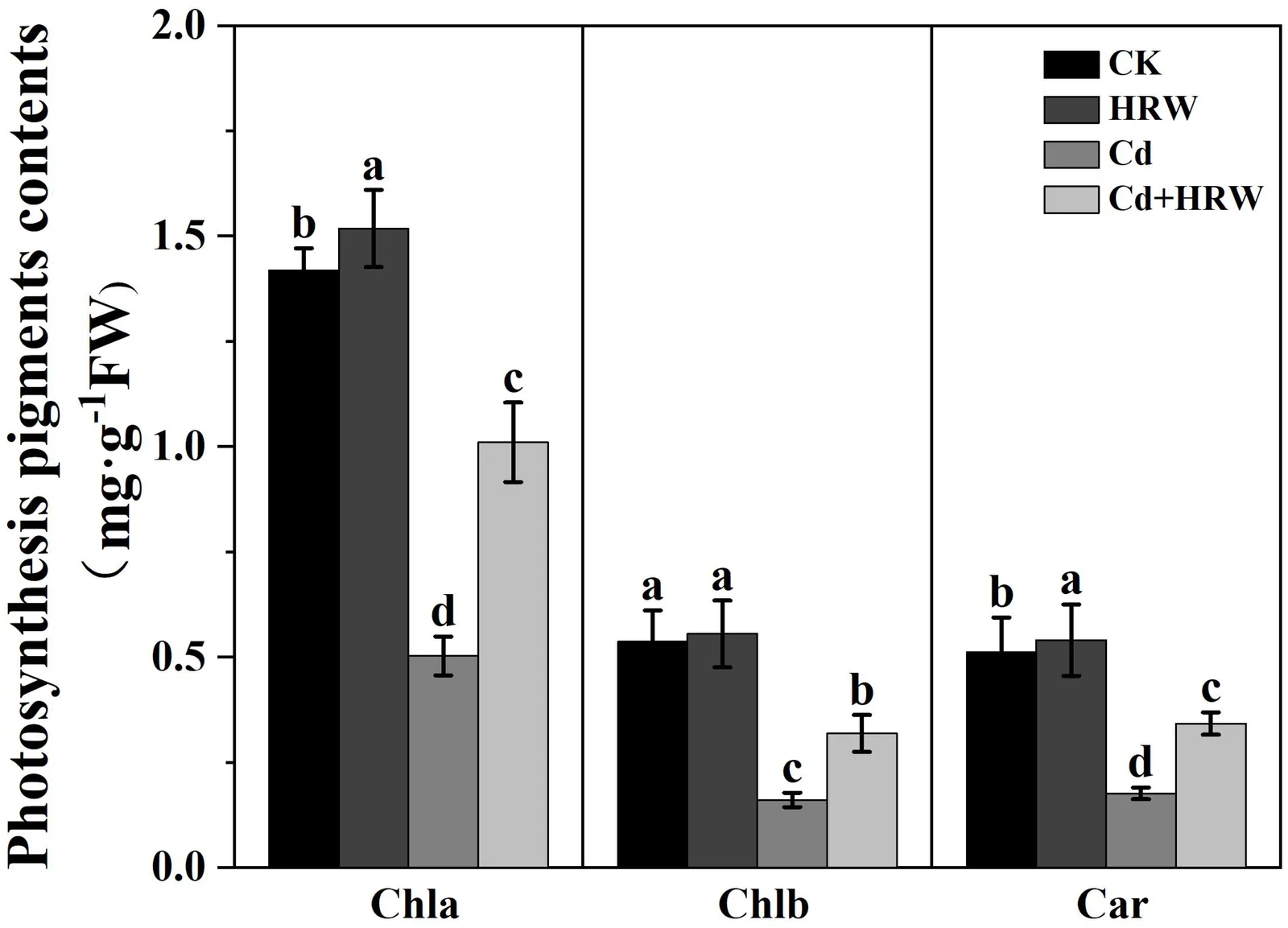
Effects of HRW treatment on leaf pigment contents in Chinese flowering cabbage under Cd stress: Chla represents chlorophyll a, Chlb represents chlorophyll b, and Car represents carotenoids. Values are expressed as the mean ± standard error (n = 10). Columns marked with different letters indicate significant differences among treatments based on the LSD test (p < 0.05).

Similarly, HRW supplementation under Cd stress enhanced the content of photosynthetic pigments (**Figure 3**). Specifically, chlorophyll a, chlorophyll b, and carotenoid contents of the Cd+HRW treatment increased by 36.76%, 29.45%, and 32.44%, respectively, compared to the Cd treatment alone. These results demonstrated that HRW mitigated Cd-induced damage to leaf pigments in Chinese flowering cabbage seedlings.

### 3.3 The effect of HRW on MDA and ROS content in Chinese flowering cabbage under Cd stress

**Figure 4:**
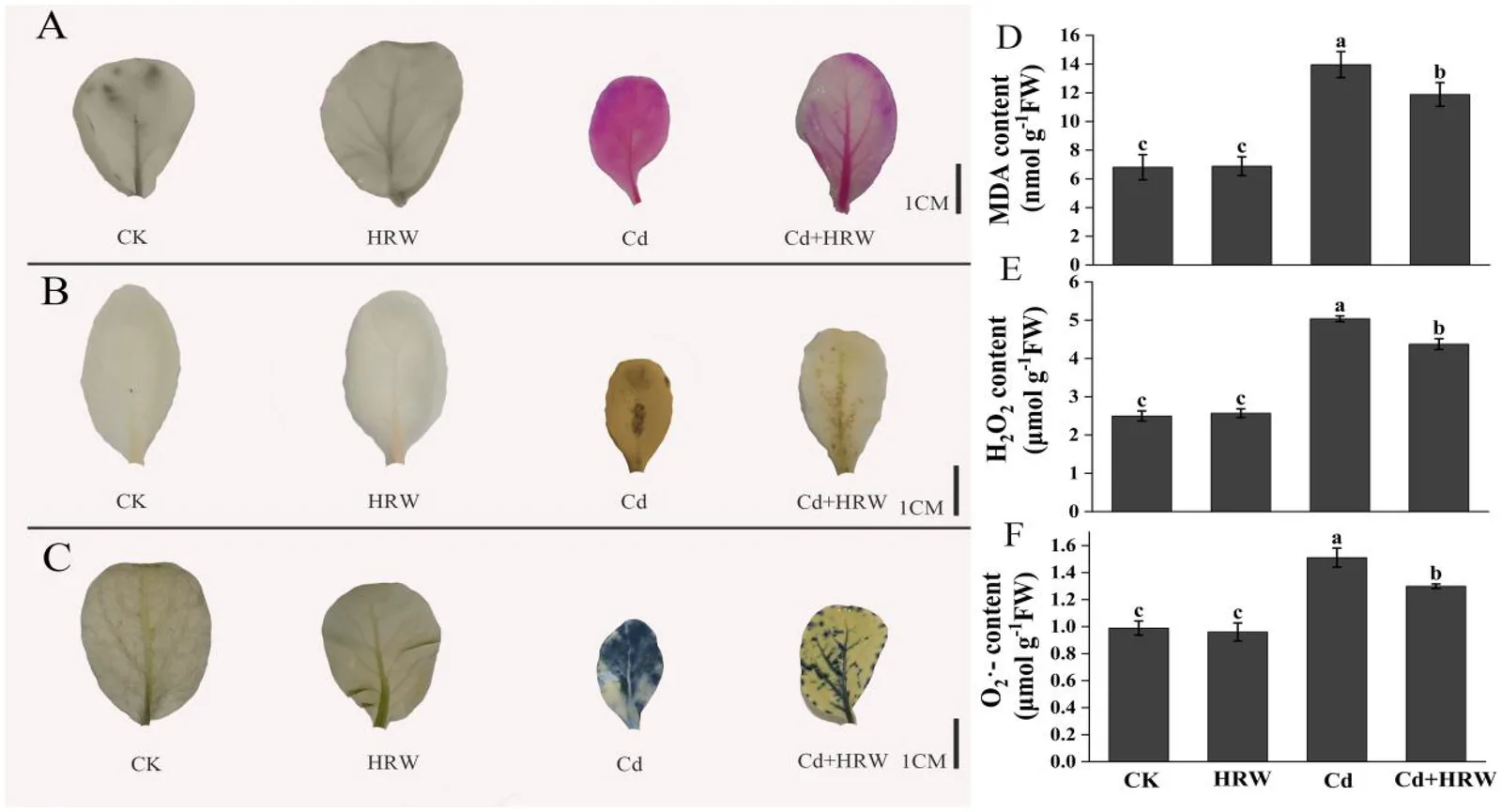
Effects of HRW treatment on MDA and ROS contents of Chinese flowering cabbage under Cd stress: **(A)** Lipid peroxidation staining diagram, **(B)** H_2_O_2_ staining diagram, **(C)** O_2_^-^ staining diagram. **(D)** changes in MDA content, **(E)** changes in H_2_O_2_ content, **(F)** changes in O_2_^-^ content. Values are expressed as mean ± standard error (n = 10). Columns marked with different letters indicate significant differences among treatments based on the LSD test (p < 0.05).

Cd stress significantly increased the accumulation of oxidative stress in Chinese flowering cabbage, including MDA (**Figure 4A**), H_2_O_2_ (**Figure 4B**), and O_2_^−^ (**Figure 4C**). However, the exogenous addition of HRW effectively alleviated Cd-induced oxidative damage, reducing MDA (**Figure 4D**), H_2_O_2_ (**Figure 4E**), and O_2_^−^ (**Figure 4F**) levels by 30.61%, 26.58%, and 21.51%, respectively, compared to Cd treatment alone. These results demonstrate that HRW plays a crucial protective role against Cd-triggered oxidative stress in Chinese flowering cabbage.

### 3.4 The effect of HRW on antioxidant enzymes and nonenzymatic antioxidants in Chinese flowering cabbage under Cd stress

**Figure 5:**
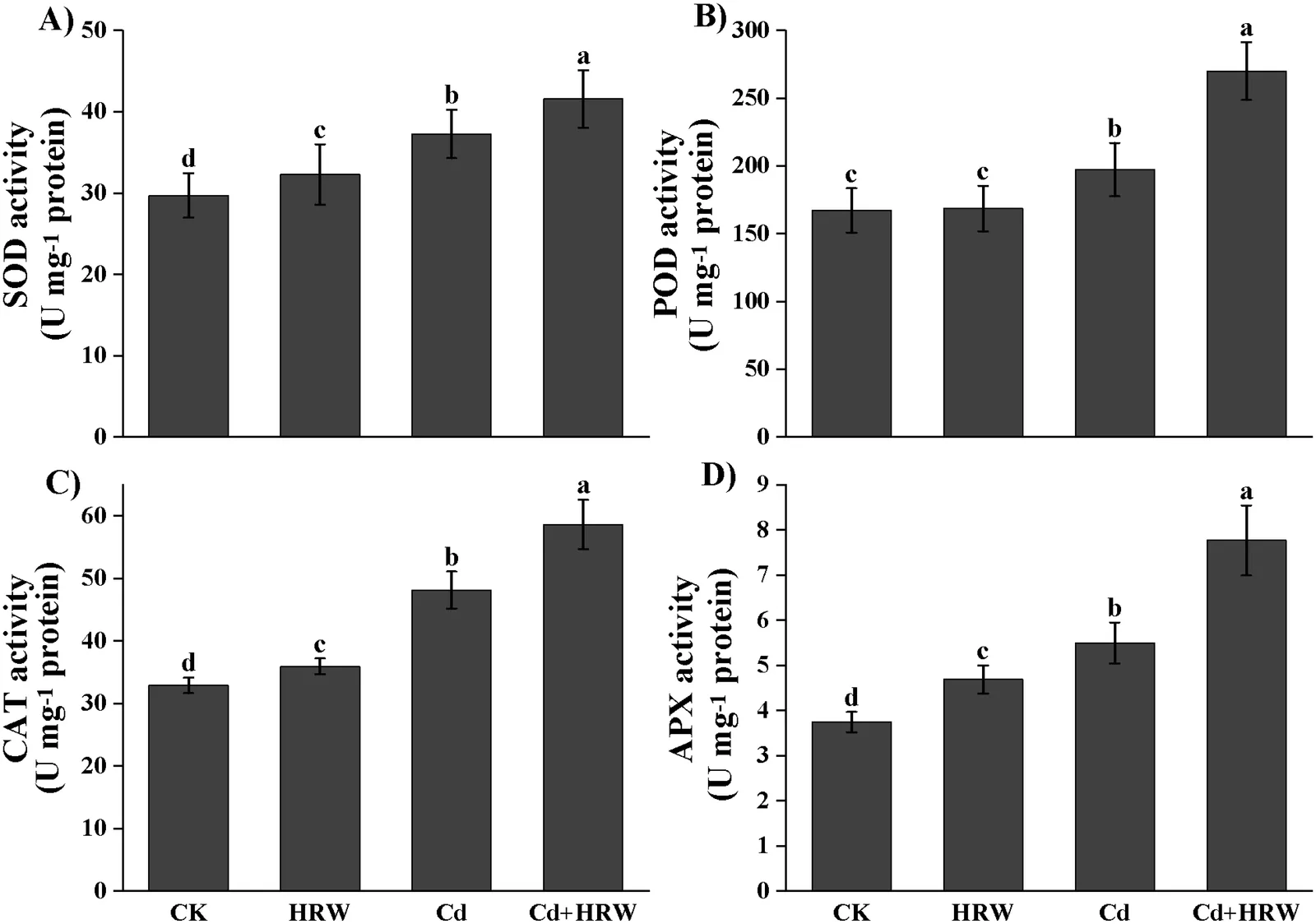
Effects of HRW treatment on antioxidant enzyme activity of Chinese flowering cabbage under Cd stress: **(A)** changes in SOD activity, **(B)** changes in POD activity, **(C)** changes in CAT activity, **(D)** changes in APX activity. Values are expressed as the mean ± standard error (n = 10). Columns marked with different letters indicate significant differences among treatments based on the LSD test (p < 0.05).

Under Cd stress, the activities of key antioxidant enzymes (SOD, POD, CAT, and APX) in Chinese flowering cabbage were significantly increased. The exogenous addition of HRW further enhanced the SOD (**Figure 5A**), POD (**Figure 5B**), CAT (**Figure 5C**), and APX (**Figure 5D**) activities by 14.52%, 43.54%, 32.11%, and 54.34%, respectively, compared to the Cd treatment alone.

Similarly, Cd stress markedly increased the contents of nonenzymatic antioxidants GSH and AsA. HRW supplementation under Cd stress further significantly elevated GSH and AsA levels by 37.14% and 22.31%, respectively.

### 3.5 The effect of HRW on Cd content, translocation coefficients, and concentrations of other metal ions in Chinese flowering cabbage under Cd stress

**Figure 6:**
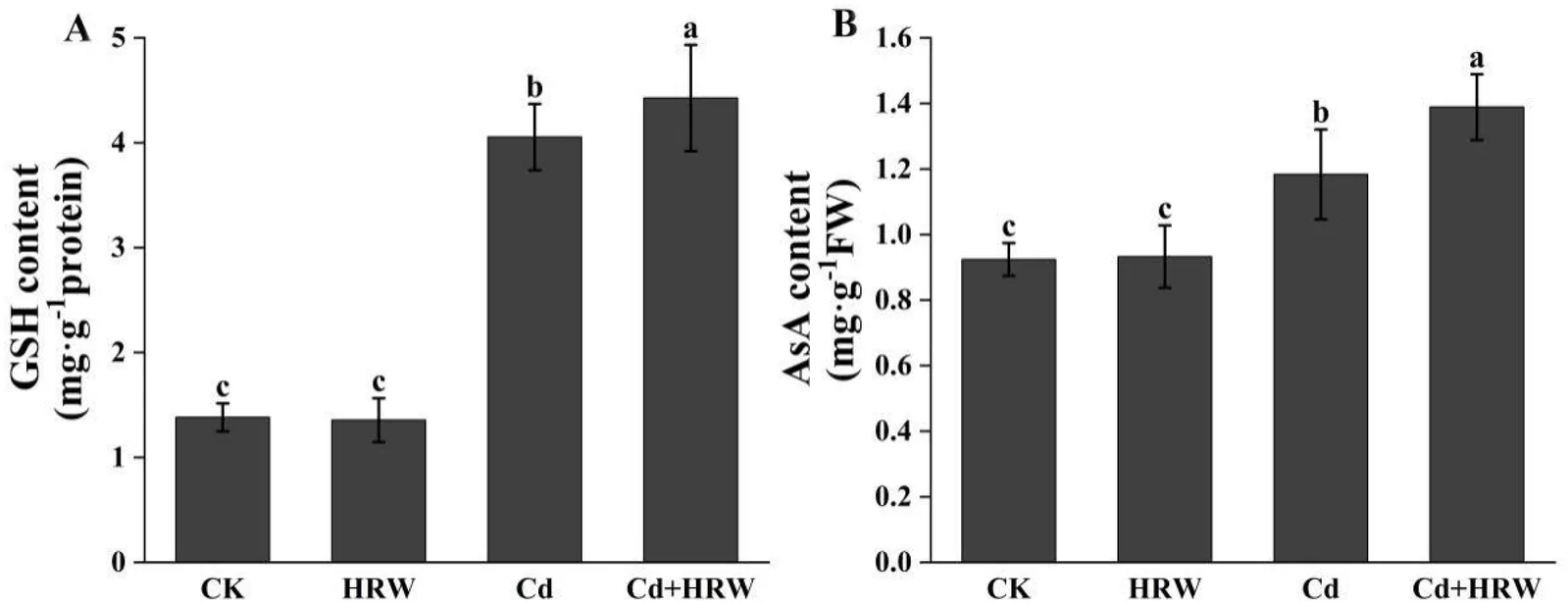
Effects of HRW treatment on Cd content in the shoot and root parts of Chinese flowering cabbage. **(A)** Changes in Cd ion concentrations, **(B)** Cd ion translocation coefficient. Values are expressed as the mean ± standard error (n = 3). Columns marked with different letters indicate significant differences between treatments based on the LSD test (p < 0.05).

The exogenous addition of HRW significantly reduced Cd accumulation in both shoots and roots in Chinese flowering cabbage under Cd stress. Compared to Cd treatment alone, the Cd+HRW treatment decreased Cd content in shoots and roots by 24.33% and 22.31%, respectively (**Figure 6A**). The translocation coefficients for Cd were not statistically significantly different, 35.68% for the Cd treatment and 36.19% for the Cd+HRW treatment, indicating that HRW had no significant effect on Cd translocation (**Figure 6B**).

**Figure 7:**
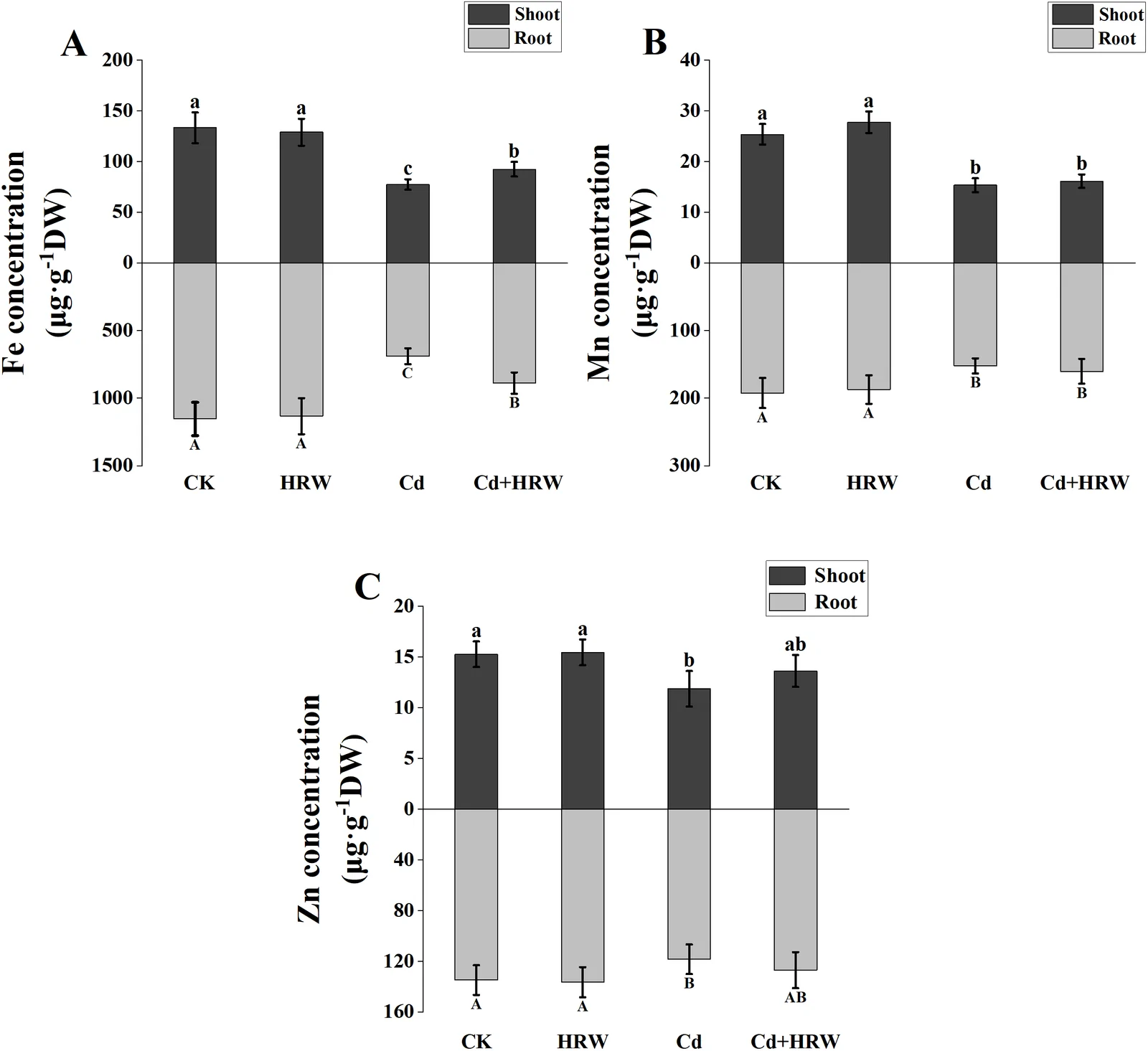
Effects of HRW treatment on changes in metal ion content in Chinese flowering cabbage. **(A)** Iron ion content, **(B)** Mn ion content, **(C)** Zn ion content. Values are expressed as the mean ± standard error (n = 9). Columns marked with different letters indicate significant differences among treatments based on the LSD test (p < 0.05).

Regarding essential mineral elements, Cd treatment significantly reduced the Fe, Mn, and Zn content compared with CK. HRW supplementation under Cd stress partially restored Fe homeostasis. The Cd+HRW treatment increased Fe content in shoots and roots by 11.34% and 17.21%, respectively, compared to Cd treatment alone (**Figure 7A**). Besides, the Mn and Zn contents under the Cd+HRW treatment showed no significant difference compared to the Cd treatment (**Figures 7B, C**).

### 3.6 The effect of HRW on the expression levels of genes related to Cd transport and uptake in Chinese flowering cabbage under Cd stress

**Figure 8:**
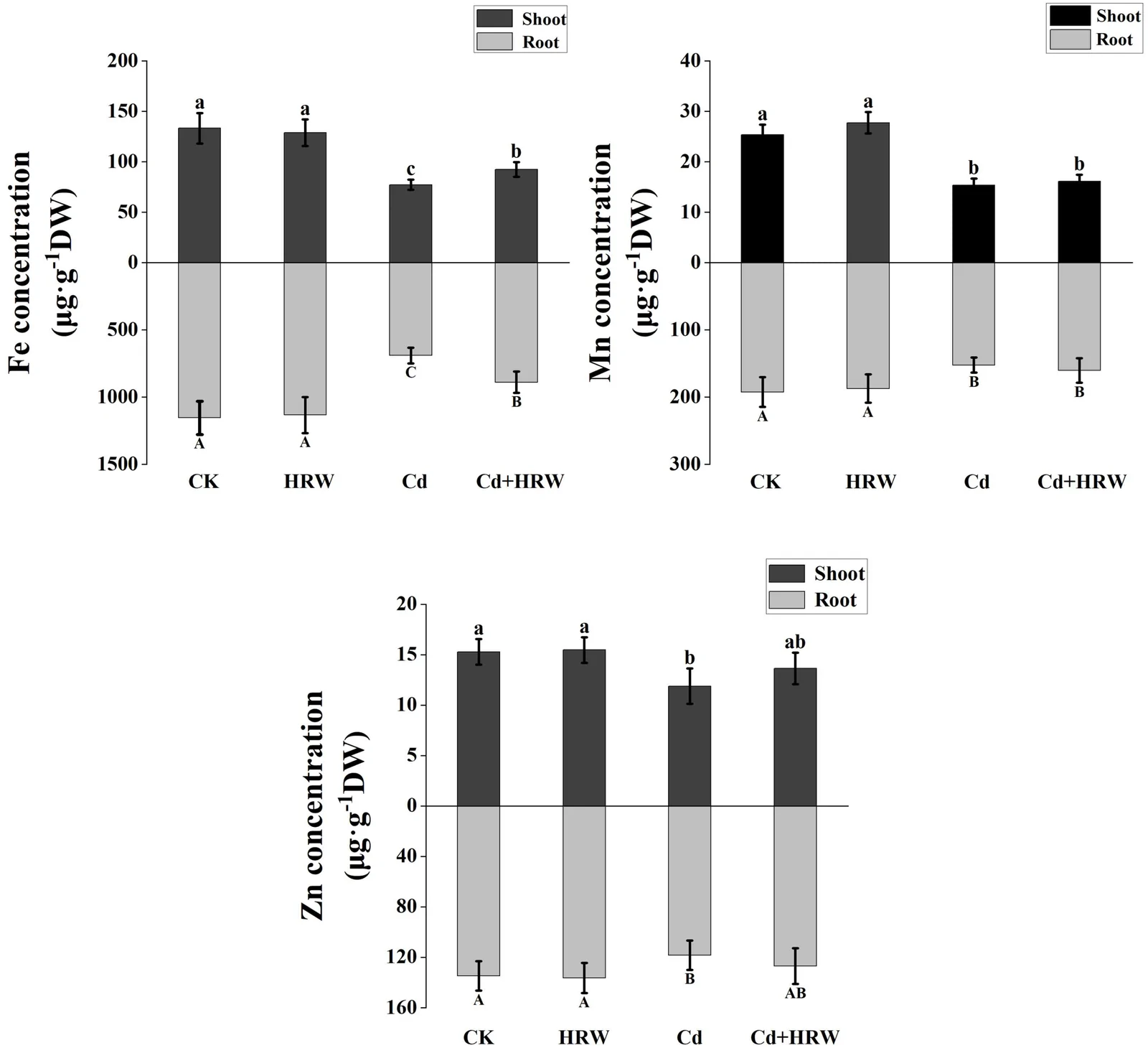
The effect of HRW on the expression levels of genes related to Cd transport and uptake in Chinese flowering cabbage under Cd stress. The expression levels of *BcIRT1* **(A)**, *BcIRT2* **(B)**, *BcNramp1* **(C)**, *BcZIP2* **(D)**, *BcABCC1* **(E)**, *BcABCC2* **(F)**, *BcHMA2* **(G)**, *BcHMA3* **(H)**, and *BcHMA4* **(I)** are shown. Values are expressed as the mean ± standard deviation (SD) of three technical replicates from three independent biological replicates (n = 9). Different lowercase letters above the bars indicate significant differences among treatments based on the least significant difference (LSD) test at p < 0.05.

The experimental results showed that, compared with the CK group, in Chinese cabbage seedlings treated with Cd alone, the expression levels of absorption-related genes (*BcIRT1, BcIRT2, BcNramp1, BcZIP2*) were significantly increased by 54.57%, 60.90%, 47.24%, and 37.03%. When treated with Cd + HRW, the relative expression levels of *BcNramp1* and *BcZIP2* were significantly decreased by 18.37% and 19.48% compared with the Cd treatment (**Figures 8A–D**). For genes such as *BcABCC1, BcABCC2, BcHMA2, BcHMA3*, and *BcHMA4*, there was no significant difference in relative expression levels among different treatments (**Figures 8E–I**). These results suggest that HRW alleviates Cd stress in Chinese flowering cabbage primarily by downregulating the expression of Cd uptake-related transporter genes *BcNramp1* and *BcZIP2*, thereby reducing Cd absorption. Meanwhile, the lack of a significant effect on the expression of Cd translocation-related genes indicates that the mitigating effect of HRW is attributed to the inhibition of Cd uptake rather than the alteration of internal Cd transport.

## 4 Discussion

### 4.1 HRW significantly improves growth and photosynthesis in Chinese flowering cabbage under Cd stress

As a highly toxic heavy metal, Cd results in the inhibition of plant growth and the disruption of their physiological functions (Gu et al., 2022). Our results indicated that Cd treatment led to a decrease in the plant height and above-ground fresh weight of Chinese flowering cabbage. In contrast, HRW significantly alleviated the growth inhibition caused by Cd (**Figure 1**). This finding aligns with HRW mitigating the growth inhibition of Cd-stressed alfalfa seedlings (Cui et al., 2020). Our findings revealed a marked reduction in root length and root fresh weight in Chinese flowering cabbage under Cd stress. Relative to the Cd treatment, the Cd+HRW treatment resulted in significant increases in root length and root fresh weight of 29.50% and 63.32%, respectively (**Figure 1**). Such severe damage to root development likely impairs the root system’s physical capacity to maintain balanced nutrient and water absorption. Consistent with this mechanistic understanding, our results demonstrate that hydrogen-rich water (HRW) treatment mitigates these stress-induced physiological and growth imbalances, likely by restoring root architecture and function (Li et al., 2019; Jiang et al., 2023). After supplementing with HRW, root fresh weight and root length were markedly increased (**Figure 1**), in agreement with studies demonstrating enhanced root growth following stress-alleviating treatments (Bushra et al., 2024).

In addition to improving root growth, the alleviating effect of HRW on plant abiotic stress is mainly reflected in its impact on the photosynthetic system. The data revealed that Cd exposure led to a reduction in photosynthesis-related parameters in the leaves of Chinese flowering cabbage seedlings (**Figures 2**, **3**). The incorporation of HRW augmented the photosynthetic efficiency and the chlorophyll concentration in Chinese flowering cabbage subjected to Cd stress, thus counteracting the photosynthetic impairment induced by Cd stress (**Figures 2**, **3**). This outcome aligns with previous studies on different plant species under Cd stress. For instance, in cucumber plants, HRW was shown to alleviate Cd-induced photosynthetic inhibition by enhancing chlorophyll synthesis and protecting the photosynthetic apparatus (Jiang et al., 2023). Similarly, the application of arbuscular mycorrhizal fungi (AMF) in Cd-stressed chickpea seedlings significantly improved photosynthetic efficiency by upregulating key photosynthetic enzymes and reducing oxidative damage (Bushra et al., 2024). In rice, foliar silicon supplementation increased the net photosynthetic rate under Cd toxicity by stabilizing chloroplast structure and enhancing stomatal conductance (Huang et al., 2023). Additionally, exogenous melatonin application improved photosynthesis in Cd-exposed safflower and cucumber by mitigating reactive oxygen species accumulation and preserving photosystem II activity (Shah et al., 2020; Amjadi et al., 2021). Therefore, the observed mitigation of Cd stress in Chinese flowering cabbage following HRW treatment may similarly involve protective effects on the photosynthetic machinery, such as enhanced chlorophyll content, reduced oxidative stress, and improved electron transport efficiency.

### 4.2 MDA, ROS levels, and antioxidant enzyme activity, and the nonenzymatic antioxidant system

Cd causes lipid peroxidation and generates ROS, leading to significant oxidative stress in plants (Wang and Yang, 2022; Altaf et al., 2023; Danilova et al., 2023). Our results showed that Cd treatment significantly increased the levels of MDA, H_2_O_2_, and O_2_^−^ in the roots of Chinese flowering cabbage seedlings, whereas HRW treatment under Cd stress decreased the accumulation of these substances (**Figure 4**). To counteract the oxidative damage caused by Cd stress, plants have developed a comprehensive antioxidant system that includes antioxidant enzymes and nonenzymatic antioxidants (Cai et al., 2023; Luo et al., 2024).

SOD is the first line of defense against ROS, catalyzing the conversion of superoxide anions into H_2_O_2_ and O_2_^−^ (Saqib et al., 2023). POD and CAT play crucial roles in scavenging H_2_O_2_ in plants (Li et al., 2023; Niu et al., 2023). Our research showed that Cd stress significantly improved the activities of SOD, POD, CAT, and APX (**Figure 5**), consistent with previous reports (Rady et al., 2023; Tang et al., 2023). Zhang et al. (2020) also reported that HRW mitigated Cd-induced oxidative stress in rice by enhancing antioxidant enzyme activities. The increase in antioxidant enzyme activities may be beneficial to the decrease in ROS accumulation (Ulusu et al., 2017). The protective effect of HRW on Chinese flowering cabbage seedlings may be related to the increased activities of antioxidant enzymes, including SOD, POD, and CAT (**Figure 5**).

**Figure 9:**
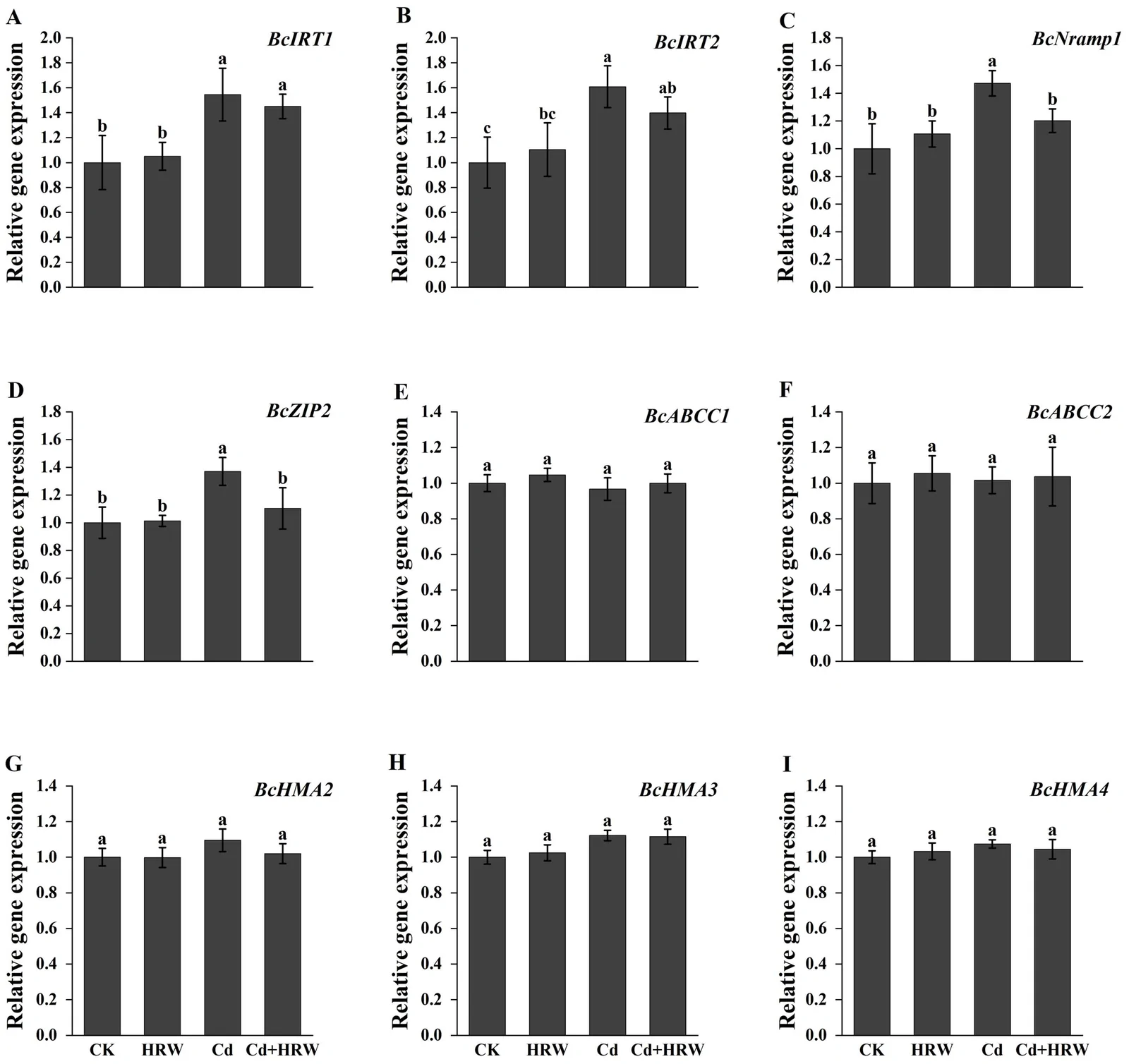
Effects of HRW treatment on AsA and GSH contents of Chinese flowering cabbage under Cd stress: **(A)** changes in GSH content, **(B)** changes in AsA content. Values are expressed as the mean ± standard error (n = 10). Columns marked with different letters indicate significant differences among treatments based on the LSD test (p < 0.05).

Furthermore, nonenzymatic antioxidants such as AsA and GSH are essential in reducing ROS-induced oxidative stress (Fu et al., 2011). In our study, compared with Cd treatment alone, HRW+Cd treatment increased the levels of AsA and GSH (**Figure 9**), which is consistent with previous research (Foyer et al., 1997), indicating that HRW alleviated oxidative damage caused by Cd stress through increased GSH and AsA concentrations (Muneer et al., 2014).

### 4.3 Cd content in relation to other metal ion levels

Cd as a heavy metal pollutant disrupts the homeostasis of essential elements in plants. Therefore, it has long been believed that the toxicity of Cd to plants is partly achieved through disturbing basic element metabolism (Muneer et al., 2014; Slamet-Loedin et al., 2015; Paradisone et al., 2021; Lu et al., 2024; Tang et al., 2024; Yao et al., 2024). Iron and zinc are critical components of many enzymes and signaling molecules, and deficiencies in these metals can affect normal plant physiological functions, such as photosynthesis and stress resistance (Li et al., 2017; Wang et al., 2024). From **Figure 7**, it could be seen that Cd treatment led to a decrease in Fe, Mn, and Zn levels. While HRW+Cd treatment increased Fe content, it had no significant effect on Mn and Zn concentrations, similar to the experimental results of other researchers (Wu et al., 2019).

Numerous studies have shown that many transporter genes are involved in the uptake and translocation of Cd in plants. Members of the ZIP family, such as *IRT1*, *IRT2*, and *ZIP2*, are important transporters for Cd uptake by plant roots (Takahashi et al., 2011; Luo et al., 2016). In addition, *Nramp1* is also considered to be involved in the internal uptake of Cd in plants (Molassiotis et al., 2006). In this study, the transcript levels of *BcIRT1*, *BcIRT2*, *BcNramp1*, and *BcZIP2* in the roots of Chinese flowering cabbage seedlings under Cd stress were increased (**Figure 8**). However, HRW treatment significantly inhibited the transcriptional level of *BcNramp1* and *BcZIP2* induced by Cd (**Figure 8**). Recent mechanistic studies have revealed that Cd uptake regulation involves key metal transporter genes. For example, in *Arabidopsis thaliana*, *HY1* overexpression was found to suppress *IRT1* expression under Cd stress, thereby limiting Cd absorption by competitively inhibiting iron transport pathways (Han et al., 2014). Li et al (Chen et al., 2017) further demonstrated that HRW reduced Cd accumulation in Arabidopsis roots by regulating heavy metal transporters. Our experimental results indicated that exogenous addition of HRW can reduce Cd content in Chinese flowering cabbage (**Figure 6**), which may be linked to the downregulation of homologous metal transporter genes, including *BcIRT1*, *BcIRT2*, *BcZIP2*, and *BcNramp1*. These findings suggest that HRW, like HY1 in Arabidopsis, may modulate the expression of conserved metal transport systems to reduce Cd uptake in plants. The relationship between HRW and these transporters needs to be further explored, and more molecular evidence is needed to fully understand the specific mechanisms by which HRW improves Cd stress tolerance in Chinese flowering cabbage.

## 5 Conclusions

Our research has demonstrated that the application of HRW effectively alleviated the adverse effects of Cd stress on the growth of Chinese flowering cabbage seedlings and notably reduced Cd levels in their edible parts. This beneficial impact can be associated with the capacity of HRW to boost photosynthesis and antioxidant defenses in Chinese flowering cabbage seedlings when subjected to Cd stress. Moreover, HRW was found to inhibit the expression of genes pertinent to Cd uptake in the roots of Chinese flowering cabbage seedlings, consequently curbing Cd accumulation. These outcomes provided a solid theoretical basis for the development of new technology to reduce Cd accumulation in edible parts of vegetable crops.

## Data Availability

The original contributions presented in the study are included in the article/supplementary material. Further inquiries can be directed to the corresponding authors.
